# Expanding the Trauma-Informed Care Measurement Toolkit: An Evaluation of the Attitudes Related to Trauma-Informed Care (ARTIC-45) Scale with SUD Workers in PIMH

**DOI:** 10.3390/bs13060471

**Published:** 2023-06-05

**Authors:** Alicia Mendez, Emily A. Bosk, Amanda Keller, Abigail Williams-Butler, Tareq Hardan, Debra J. Ruisard, Michael J. MacKenzie

**Affiliations:** 1 School of Social Work, Rutgers University, New Brunswick, NJ 08901, USA; emily.bosk@ssw.rutgers.edu (E.A.B.); aw643@ssw.rutgers.edu (A.W.-B.); 2 School of Social Work, McGill University, Montreal, QC H3A 1B9, Canada; amanda.keller@mail.mcgill.ca (A.K.); tareq.hardan@mail.mcgill.ca (T.H.); michael.j.mackenzie@mcgill.ca (M.J.M.); 3 The Center for Great Expectations, Somerset, NJ 08873, USA; debbie.ruisard@gmail.com

**Keywords:** trauma-informed care, trauma, trauma-informed organization, service delivery, psychometric analysis, adverse childhood experiences

## Abstract

Human service organizations (HSO) have increasingly recognized the value of employing trauma-informed care (TIC) in a variety of practice settings. Evidence suggests that effectively adopting TIC has shown client improvements. Organizational barriers to TIC implementation, however, exist. To improve TIC practice, the attitudes related to trauma-informed care (ARTIC) scale was developed to measure staff attitudes and beliefs towards TIC. The ARTIC has been widely adopted by researchers without evaluating its psychometric performance in diverse practice settings. The purpose of this study was to independently validate the ARTIC scale drawn from a sample of staff (n = 373) who provide services to substance-using parents. Psychometric tests were conducted to evaluate how the ARTIC performs with our HSO population. Results from a confirmatory factor analysis showed poor fit (*X*^2^ = 2761.62, *df* = 2.96; RMSEA = 0.07 [0.07, 0.08]; CFI = 0.72). An exploratory factor analysis was conducted to analyze how the data fit with our specific population, yielding 10 factors. Finally, a qualitative inter-item analysis of these factors was conducted, resulting in nine factors. Our findings suggest that measuring TIC attitudes and beliefs may vary according to field of practice and ethno-racially diverse workers. Further refinement of the ARTIC may be necessary for various services domains.

## 1. Introduction

Experiences of trauma are common, impacting as many as 50–70% of people worldwide [1,2,3]. Understandings of the negative impact of adverse childhood experiences (ACEs), especially when compounded by environmental adversity, have led to calls for mental health and social services to be trauma-informed [4,5]. Responding to this call, human service organizations (HSOs) have increasingly pivoted toward implementing trauma-informed care (TIC) models across human service domains of practice over the last decade [5,6,7]. 

Trauma-informed care (TIC) is a widely accepted framework characterized by being mindful of traumatic experiences and their impact, creating safe environments, prioritizing clients’ voices to guide treatment, and flexibility [8,9,10,11]. As TIC interventions spread within HSOs, so too did the need for frontline workers to attain the skills, knowledge, and support necessary for transforming organizations into ones that are meaningfully “trauma-informed” [12,13,14]. While the core principles of TIC are clear, confusion remains about how to operationalize these principles in everyday practice, resulting in increased need for TIC approaches to be better routinized and operationalized in their delivery [5,15,16,17,18,19]. 

Validated tools measuring attitudes and practices related to TIC are an important component of closing the implementation gap that currently exists across programs [4,16,20,21]. The attitudes related to trauma-informed care (ARTIC [18]) scale is a validated measure of attitudes and beliefs of frontline staff about TIC created to fill this gap. The ARTIC has three versions of varying lengths; the ARTIC-45 with 45 items, the ARTIC-35 with 35, and the ARTIC-10 with 10 items. The measure targets those working in human services organizations, and those who work in schools [19,20]. Following its dissemination in 2016, there was a rapid uptake of the ARTIC. There are more than 250 citations of the original psychometric development article [20], many of which measure the extent to which individuals are trauma-informed. 

A recent scoping review of TIC measures by Wathen and colleagues [19] showed that of existing TIC measures, the ARTIC is the only one that is well-cited and used in HSOs across domains of practice. Despite its widespread use, there has been almost no research that evaluates how the ARTIC performs in specific HSO spanning contexts as diverse as criminal justice, medicine, child welfare, or addiction. Two groups, however, have evaluated how the ARTIC performed with their populations including nurses (ARTIC-35) [22] and Japanese healthcare workers (ARTIC-10) [23]. Baker and colleagues [24] also replicated their validation of the ARTIC on a new sample including 888 participants who work in education settings and 507 participants who work in human services/health settings, showing strong internal consistencies and support of construct validity. However, similar with their original sample, participants were predominantly white (75.2%). Due to the flexible nature embedded in TIC practices [8,11], it is necessary to examine how the ARTIC, and other TIC measures [25] perform with different worker populations across professional disciplines and fields of practice. Of equal importance is testing how the ARTIC performs with members of different racial and ethnic backgrounds.

It is unclear why the ARTIC has been rapidly utilized in human service organizations without additional testing in other settings and among different populations. Outside of the ARTIC’s creators, no other validation of the ARTIC-45 exists to the authors’ knowledge. The existing external validation [22,23] articles that do exist suggest that the ARTIC-35 and ARTIC-10 need further refinement. This article seeks to build on this growing body of work by evaluating how the ARTIC-45 performs in human service organizations that focus on maternal and early childhood mental health and parental substance-use disorder (SUD) recovery who are racially and ethnically diverse.

## 2. Literature Review

### 2.1. TIC in Practice

Effective TIC practices improve care by minimizing the re-traumatization of individuals within human service organizations [19,25,26]. TIC seeks to understand and place each individual seeking care within their cultural and historical background [27,28]. By embedding TIC practices within organizations, the goal is to have all levels of staff speak a common language and utilize a standard set of practices to ameliorate the effects of trauma within the specific care being sought [8,11]. TIC has the potential to transform services. Human services organizations that have effectively adopted TIC have shown changes related to increased worker understanding of TIC, positive attitudinal changes toward TIC, and greater use of TIC practices [29,30]. Moreover, when workers align their attitudes and practices with TIC, it results in improved client outcomes including symptom reduction, improved mental health, more trust and openness between client and staff, and reduced substance-use disorder (SUD) [19,31,32,33]. 

Despite the growth of TIC, multiple organizational barriers still impede effective TIC implementation [8,34]. Barriers to implementation include time constraints, a lack of necessary resources to train staff, a lack of appropriate supervision, and staff resistance to TIC principles [7,15,35,36]. Compounding these structural barriers, increasing evidence suggests that TIC is not implemented consistently across programs or organizations in large part due to the flexibility infused within trauma informed interventions [8,11,14]. Implementing TIC well is harder, requires more patience and creativity in practice, and is more demanding on workers when compared to more punitive modalities such as therapeutic community programs [15,37]. 

### 2.2. Introduction to the ARTIC

As one of the first scales to measure attitudes and beliefs about TIC, the attitudes related to trauma-informed care (ARTIC) has been widely adopted by researchers and evaluators examining the implementation and effectiveness of TIC [19]. The ARTIC was developed using a validation process that began with building upon the risking connection scale, which measured trauma-related beliefs and attitudes [28,38]. Baker and colleagues [20] presented 75 items to an expert panel for feedback. The scale uses a bipolar Likert-type scale that includes response options that are opposite extremes of the same construct [39]. Scale points are presented between the two items. Using items as the “poles” in a Likert-type scale has been shown to minimize social desirability bias [40]. 

Three versions of the ARTIC were created, the ARTIC-45, 35, and 10, with the number indicating how many items the scale included. Normative data established by the ARTIC’s creators show strong construct and criterion-related validity [20,24]. Each version then underwent a confirmatory factor analysis, showing a good model fit [20]. Internal reliabilities were measured across all items and subscales, with results indicating strong internal consistency of the seven-factor ARTIC-45 (α = 0.93), the five-factor ARTIC-35 (α = 0.91), and the abbreviated single-factor ARTIC-10 (α = 0.82) [20]. Finally, a test-retest was conducted with 141 of the original 760 participants, with results indicating support for temporal consistency [20]. 

While this scale has provided valuable information about these processes, it is worthwhile noting that the sample utilized in establishing the psychometric properties of the ARTIC predominantly comprised white college-educated staff [20,24]. There are two studies that have since evaluated the ARTIC’s psychometric properties on different worker populations that have resulted in different psychometric results. Results from these independent studies suggest that the use of bipolar Likert-like scale in the ARTIC has challenges: (1) some items are not mutually exclusive bipolar; (2) some items include more than one question; (3) some items measure different concepts on each extreme; and (4) use of the bipolar scale may result in those from different cultural backgrounds choosing a midpoint answer due to social desirability bias [22,23]. These results suggest that before the ARTIC is used in other contexts, additional psychometric testing and qualitative analysis of items is needed.

Implementing TIC is challenging, particularly due to its flexible nature in practice [7,11], making the evaluation of TIC interventions equally difficult [8]. More work is needed to understand how the ARTIC performs in other milieus with different populations, especially staff populations that are more heterogenous along dimensions of race, ethnicity, gender, and education. Building upon the work that examines the ARTIC’s psychometric properties, this study seeks to (1) replicate the validation of the ARTIC-45 assessment, and (2) examine its applicability in substance-use treatment settings that specialize in parent–infant mental health and addiction. By reviewing the ARTIC-45’s utility among workers in this domain of practice, this article will contribute to the growing understanding of how TIC is measured and has potential to advance ways to improve its measurement.

## 3. Methods

### 3.1. Participants

A total of 376 staff members from three human service organizations specializing in substance-use treatment and parent–infant mental health who participated in TIC trainings were invited to participate in this study, of whom 373 agreed. Demographic items were not initially included in the survey, but among 226 participants who provided demographics when they were added, 82% identified as female, 42% reported their race as non-white (16% Black; 3% Asian; 17% Latino; 6% Multiracial), and 20% identified as Hispanic, (see Table 1). Participants were also highly educated with 72% having completed a college degree or greater. Regarding income, 36% of participants reported a household income of USD 40,000 or less, 42% between USD 40–USD 60,000, and 21% reported USD 60,000 or more. Finally, 54% of participants reported previous trauma training.

### 3.2. Procedures

Data were collected from frontline staff, supervisors, and administrators of three human service organizations located in the Northeastern United States. Participating organizations were part of a trauma-informed intervention, the attachment, regulation and competency (ARC) model, aimed at improving attachment experiences for individuals [41]. ARC emphasizes flexibility rather than implementing a highly structured, manualized protocol [41]. Three initial ARC trainings occurred periodically over the course of three years as organizations joined the intervention. Trainings were led by the same two ARC trainers. Prior to each training, staff were invited to complete an online survey using Qualtrics. The survey included the ARTIC-45 and other scales related to job satisfaction and attitudes about place of work. The survey took approximately 45–60 min to complete, and staff received no incentive. The study was approved by the university’s Institutional Review Board and participants provided informed consent.

### 3.3. Measures

The ARTIC-45 measures staffs’ beliefs and attitudes about TIC. It contains seven subscales, with the first five “core” subscales of (1) underlying causes of problem behavior and symptoms, (2) responses to problem behavior and symptoms, (3) on-the-job behavior, (4) self-efficacy at work, and (5) reactions to the work. The last two subscales are considered “supplementary” and include (6) personal support of TIC and (7) system-wide support for TIC [20]. The five core subscales include seven items each and the two supplementary subscales consist of five items each [20]. The scale uses a 7-point bipolar Likert-like scale, where each item has two opposing responses representing either a favorable or unfavorable attitude toward TIC, or a TIC-indicated and TIC-contraindicated intervention (e.g., “I don’t have what it takes to help my clients” and “I have what it takes to help my clients”) [20].

### 3.4. Data Analysis

#### 3.4.1. Quantitative

To assess the ARTIC-45’s psychometric properties with workers in substance-use disorder facilities that focus on parent–infant mental health, we first examined validity and reliability by conducting an item analysis, internal reliability checks, and confirmatory factor analysis (CFA). We tested for internal reliability by conducting an intra-item correlation and testing for Cronbach’s alpha. Modeled after Baker and colleagues’s work [20], we performed the item analysis by examining how the items within each subscale correlated with one another. Additionally, subscale totals were examined to see if they also correlate with the ARTIC composite score. A minimum score of 0.3 for intra-item correlation is considered acceptable, with higher correlations indicating a stronger link between items and their construct [39]. We then calculated Cronbach’s alpha for the full scale (45 items), and its seven previously created subscales. We adopted DeVellis’ [39] alpha acceptability guidelines, which considers alpha below 0.7 to be unacceptable, between 0.7 and 0.8 respectable, between 0.8 and 0.9 very good, and more than 0.9 implies that the scale could be shortened.

A CFA is most appropriate for measures that are well-established; thus, our goal was to examine the validity of the ARTIC-45 with our specific sample [42]. Fit indices used to evaluate model fit included the chi-square (*X*^2^) test, the chi-square *X*^2^ to degrees of freedom ratio or (*X*^2^:*df*), the root mean square error of approximation (RMSEA), and the confirmatory factor index (CFI). While the chi-square *X*^2^ is often reported, it is generally not an acceptable model fit indicator as it is sensitive to sample size. Fit index values that indicate adequate CFA model fit are a nonsignificant chi-square *X*^2^ test, a chi-square to degrees of freedom ration (*X*^2^:*df*) ranging between 2 and 5, RMSEA less than 0.06 [43], and CFI greater than 0.95 [43].

Based on the CFA results, we next performed an exploratory factor analysis (EFA) using maximum likelihood estimation to examine the ARTIC-45 factor structure in our sample [44,45]. An EFA was implemented because our results and other independent validations of the ARTIC suggest that the scales’ items may not actually be measuring TIC [22,23], suggesting an exploratory approach may be needed. Maximum likelihood estimation was chosen because our data is normally distributed [46,47]. Factors with eigenvalues greater than 1 are generally considered distinct factors extracted from the EFA [42]. However, not all latent variables with eigenvalues greater than 1 may become a factor or subscale, depending on the variance explained by each factor, theoretical knowledge, model parsimony, and other considerations [42,47]. All quantitative analyses were conducted using SPSS version 26 and SPSS AMOS.

Additional sensitivity analyses were conducted. As the ARTIC-45 was created to measure seven independent constructs, a forced 7-factor EFA was conducted. Additionally, a CFA was performed on the ten items that make up the ARTIC-10 due to our quantitative results suggesting a unidimensional factor approach.

#### 3.4.2. Qualitative

Using the results from the original EFA, we conducted an inter-item qualitative analysis for each factor with items loading at 0.3 or greater to identify how the ARTIC’s items performed in a substance-use treatment setting [22]. The goal of this analysis was to qualitatively understand how the items held together by thematically analyzing each factor’s list of items and comparing the subsequent themes to the original seven factors. Four team members (authors 1, 2, 3, and 5) with expertise in TIC engaged in an analysis of the factors derived from the EFA and their corresponding items. Analysts met to discuss TIC subscale themes and choose which items, if represented on more than one factor, align more with the TIC themes present in the data. All items that corresponded with each factor were listed and qualitatively analyzed. Multiple items appeared on two or more potential factors. Factors were individually open-coded by each analytic team member to identify which theme was represented based on its items. Next, the analytic team compared their themes to come to a group consensus. Through discussion, the group then came to a consensus about naming each factor. Finalized factor themes represent complete agreement among team members. Following this process, items were deleted if agreement was not reached on whether it should be included in a subscale.

## 4. Results

### 4.1. Item Analysis

A majority of the items (4 or more) within each of the five core subscales did not correlate with one another. When examining the two supplementary subscales “personal support of TIC” and “systems-wide support for TIC”, all items correlated with fellow subscale items at 0.3 or greater. Next, in an effort to replicate the original validation, all seven subscales were correlated with one another and the ARTIC composite score. Correlation values ranged from 0.56 to 0.85, or satisfactory to strongly satisfactory.

### 4.2. Cronbach’s Alpha

The full ARTIC-45 scale’s internal consistency was strong in our data, (α = 0.98), suggesting that it could be shortened [39]. All seven scales’ alphas ranged between 0.72 and 0.81, which indicated respectable to very good scores.

### 4.3. Confirmatory Factor Analysis

**Figure 1:**
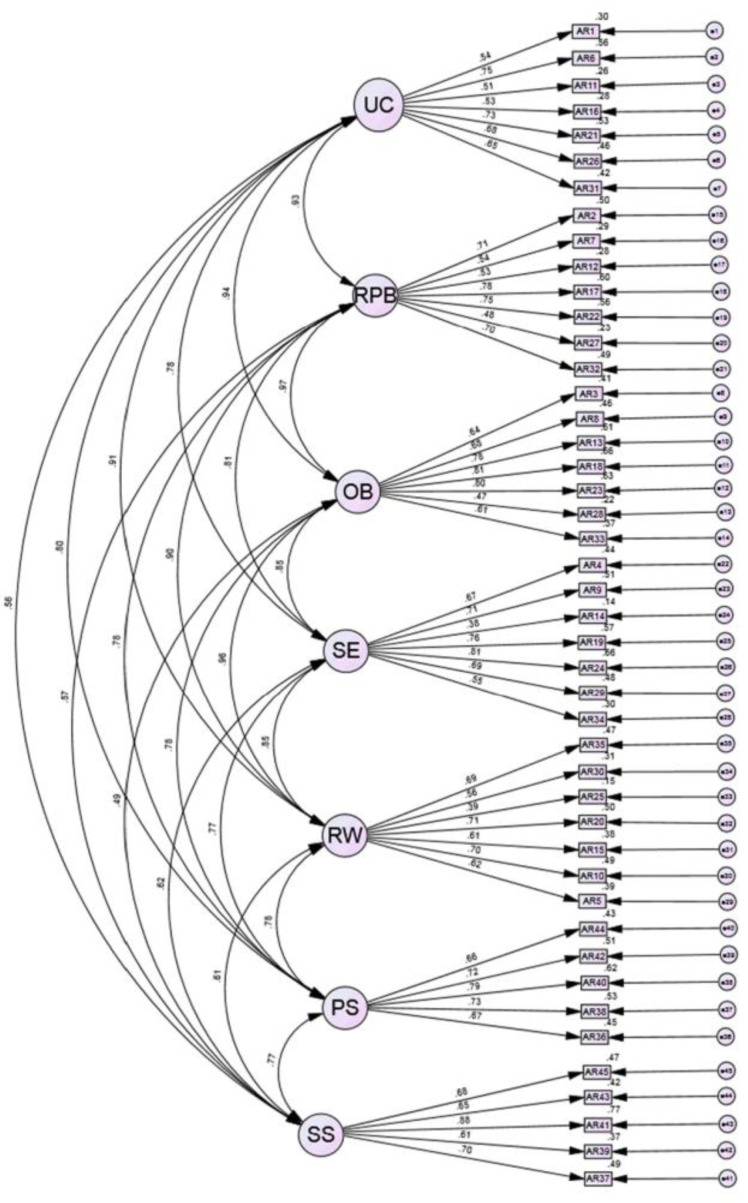
Confirmatory factor analysis results included a chi-square of *X*^2^ = 2761.62, and a *X*^2^:*df* = 2.96. The RMSEA = 0.07 [0.07, 0.08] with a CFI = 0.72 indicating a poor model fit.

Next, we conducted a confirmatory factory analysis (CFA, Figure 1) using maximum likelihood estimation. This CFA produced a chi-square of *X*^2^ = 2761.62, and a *X*^2^:*df* ratio = 2.96 (2761.62:930), suggesting a good model fit. Both the RMSEA (0.07 [0.07, 0.08]) and the CFI (0.72) indicated a poor model fit (See Figure 1).

### 4.4. Exploratory Factor Analysis and Sensitivity Analyses

**Figure 2:**
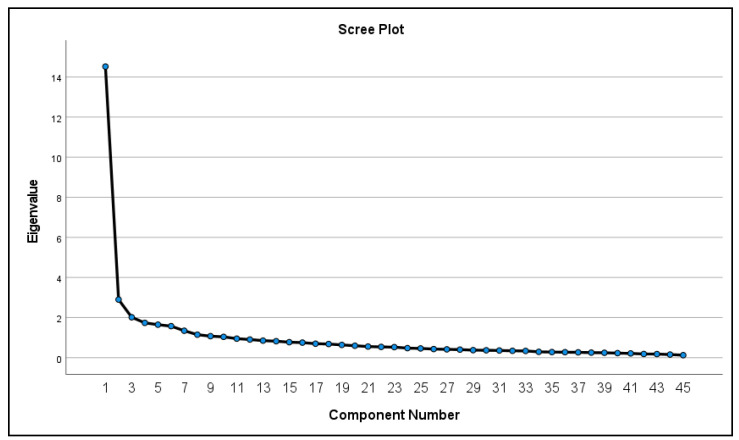
Scree plot for the EFA performed shows factors with eigenvalues equaling more than 1.

An exploratory factor analysis (EFA) using maximum likelihood estimation was conducted to assess the number of distinct ARTIC-45 factors in our sample. Our sample produced 10 eigenvalues greater than one suggesting potentially 10 factors for our data (see Figure 2). Factor 1 within the EFA loaded 43 of the 45 items with a score of 0.3 or greater. A sensitivity analysis that limited the sample to participants who provided demographic information (N = 217) produced similar results.

A forced factor EFA was conducted to analyze whether the data, when forced, recreated the same spread of items on its original seven factors. Results from both EFAs showed that 40–43 of the 45 items loaded at 0.3 or greater onto factor 1, further suggesting a unidimensional factor structure. Results from the CFA using items that make up the ARTIC-10 showed poor fit. This CFA produced a chi-square of *X*^2^ = 185.41, and a *X*^2^:*df* ratio = 5.30 (185.41:35), suggesting a poor model fit. Both the RMSEA (0.11 [0.09, 0.12]) and the CFI (0.86) indicated a poor model fit.

### 4.5. Qualitative Analysis

Initial qualitative analyses of the factors indicated that the tenth factor should be eliminated from the scale as no items had loadings of 0.3 or greater on this factor. The final nine factors included the following themes: organizational support for provision of tIc, trauma theory, reflective functioning on the job, worker efficacy, relationships with clients, efficacy of TIC, importance of consequences, beliefs about clients, and secondary traumatic stress (see Table 2). ARTIC-45 items 1, 4, and 10 were not represented in our final version of the scale as the items did not qualitatively fit with any corresponding factors. These results suggest that the ARTIC qualitatively performs different with our population.

## 5. Discussion

Building on existing work, our results indicate that additional refinement of the ARTIC is needed [19,22,23,24]. The ARTIC may perform differently with our population for three reasons: (1) workers who primarily work in an organization that specializes in substance-use disorder and parent–infant mental health may differ from the populations the original ARTIC was normed on; (2) cultural differences may result in workers responding differently to the ARTIC items; and (3) the ARTIC’s bipolar Likert-like scale may not be accurately representing two opposing poles for each item [22].

Quantitative results indicated that the ARTIC-45’s original seven factors and corresponding items did not hold up similarly with our population of frontline workers who work with substance-using populations. EFA results suggest a 10-factor scale. Upon closer examination, with this working population, the ARTIC-45 may be a unidimensional scale as more than 40 of the 45 items loaded onto factor 1. The ARTIC-10, created at the same time as other versions of the ARTIC, does represent a unidimensional scale. However, our additional CFA on items representing the ARTIC-10 did not show good model fit with our population, suggesting that more inquiry is needed to understand why the ARTIC is not performing as strong.

The ARTIC’s psychometric properties were originally examined using workers in community-based mental health and healthcare facilities and workers in education. It is possible that those working in a substance-use disorder treatment and parent–infant mental health (PIMH) support may understand and experience examples of trauma-informed care differently in these settings. Workers in these specific HSO settings are balancing multiple foci of care including substance-use recovery, individual trauma-focused work, and attempting to improve parent knowledge and their bond with their child. Often, workers in these HSOs specialize in one or two of those areas, but not always all three, possibly making it difficult to integrate a one-size-fits-all trauma-informed practice.

These differences may be due to our diverse sample (16% Black; 3% Asian; 17% Latino; 6% Multiracial; and 20% Hispanic). The ARTIC was normed on a largely white, upper- and middle-class socio-economic population (91% white [20]; 75% white [24]), raising questions about how it will perform with a more racially and class diverse group of providers and frontline workers. Kataoka and colleagues [23] tested the validity and reliability of the ARTIC on Japanese healthcare workers, showing similar issues. Their results suggest that cultural differences between the original sample compared with their own may be part of the reason the ARTIC did not perform similarly.

As with understandings of measuring psychological variables with diverse ethno-racial groups, it is possible that workers of color may interact with certain questions differently [48]. Those from varying cross-cultural backgrounds, which are different from white and Western cultures, may perceive questions differently resulting in measurement errors [49]. For example, Kataoka and colleagues [23] note that compared to people from Western cultures, Japanese people are more likely to choose a midpoint response due to social desirability bias [50]. Related, Black respondents also show evidence of social desirability bias by responding to items in a way that minimizes associations with anger or other negative emotions [51,52]. Thus, some of our sample may have answered in a way that they thought looked more accepting of TIC. However, due to the items’ wording issues, that remains unclear. It is also possible that differential item functioning may be occurring, suggesting items may need to be written differently for different groups, in this case, groups from different ethno-racial backgrounds [49,51,52]. Renewed attention to the ways in which systemic racism pervades institutional, organizational, and social life make it imperative to norm instruments related to TIC on a diverse population and to examine how issues related to positionality and dimensionality may influence responses and all aspects of validity, particularly in an employment context.

Following qualitative inter-item analysis, the nine subscales resulted in some similar and differing themes from the original ARTIC. While we do not suggest uptake of our nine-factor scale, we do suggest more inquiry into the qualitative nature of the ARTIC’s items. Aligned with previous work, we suggest that this scale being categorized as a bipolar Likert-type scale is potentially a misnomer and closer resemblance to a semantic differentiation scale [22,23]. Taken together, these results raise new questions about the phrasing of questions on the original scale and their attendant impact on results. One solution to the latter issue may be to rephrase statements so that they each reflect true polar opposites of the same dimension. This could be accomplished by slightly rewording the second statement. For example, the current polar statements of item 1: “Clients’ learning and behavior problems are rooted in their behavioral or mental health condition.” and “Clients’ learning and behavior problems are rooted in their history of difficult life events.” could be rephrased so that the second statement now reads as follows: “Clients’ learning and behavior problems are not rooted in their behavioral or mental health condition.” Following this approach, the second statement on item 3 could also be rephrased. Currently, the item reads as follows: “Being upset is normal for many of the clients I serve.” and “It reflects badly on me if my clients are very upset.” The second statement could be rephrased to “Being upset is abnormal for many of the clients I serve” in order to add further clarity to the respondents’ choices. These phrasing issues need to be resolved to have confidence that these questions best measure the constructs they intend to assess.

To date, the ARTIC has been shown to perform differently with at least three varying worker populations (nurses [22], Japanese healthcare workers [23], and the current analysis); this suggests that the overall use and application of the ARTIC needs further adjusting. We suggest that future use of the instrument also include an evaluation of its psychometric properties, and that further refinement be applied to the ARTIC’s items, rather than an entirely new instrument itself [53]. This may preserve better historical comparisons if, for example, the result is a shortened instrument containing items from the original scale; thus, new work would continue to be able to be compared to earlier datasets, which could simply be recoded to reflect any adjustments to the scale syntax. By taking these steps, it is possible the human service organizations and evaluators may draw closer to a shared operationalized understanding of TIC in practice.

## 6. Limitations

While our findings raise important questions for the future measurement of TIC attitudes, there are several limitations. The results of Cronbach’s coefficient alpha indicated strong internal consistency. However, these results may differ if we used a different approach to estimating a coefficient alpha that is more suited for a multidimensional scale [49,54]. Our paper did not take these steps in an effort to replicate the original study as closely as possible.

As Baker and colleagues [20] indicate in their initial psychometric evaluation of the ARTIC-45, further research is needed to assess how the ARTIC-45 may perform with other populations. Our results are limited to front-line staff treating SUD among parents with children. Further, while we opted to analyze how the items performed with our population, we did not explore different semantic iterations of items with workers. While we qualitatively analyzed the 10-factor EFA results, we are not suggesting the uptake of our results. Future research would benefit from sampling different alternatives to the ARTIC-45, including rewording the bipolar items to be more uniformly opposite and using a traditional Likert-scale for each of the items with choices such as strongly agree to strongly disagree.

## 7. Conclusions

As one of the first, and most widely used, tools to measures attitudes towards TIC, the ARTIC has experienced a rapid and broad uptake in the field without the necessary additional psychometric evaluation (SAMHSA, 2021; Wathen et al., 2023 [10,19]). The convergence around the ARTIC as a tool for assessing TIC makes sense given the paucity of other measurements combined with the emergent need to analyze new TIC efforts. At the same time, there has been too little examination of potential measurement issues within the ARTIC [19,53]. These findings indicate that the conditions under which the ARTIC will have the best utility should be more closely examined as it may not be accurately measuring TIC among all populations.

As the field continues to evolve and new knowledge about TIC is developed, there is also a corresponding need to consider how this new knowledge should be captured in research. For example, it is clear that while TIC enjoys a broad level of endorsement and support from frontline social service providers and mental health workers, there is little shared understanding of how the core principles of TIC should be operationalized, which has resulted in widespread variation in implementation [7,15,16].

While the ARTIC has value as one of the first TIC scales utilized in HSO settings, it is imperative for the field to continue to advance in regard to measurement to achieve a robust and rigorous set of assessments about the provision of TIC in different social service settings with different client populations. More research is needed to identify the conditions under which the ARTIC should be used and how it may perform differently across different working populations and diverse ethno-racial groups.

## Figures and Tables

**Table 1:** Sample descriptive statistics. Some questions were listed as choose all that apply.

	Mean or % (n)
Sex	
Male	18.1 (42)
Female	81.9 (190)
Race	
Black or African American	16.4 (37)
Asian	3.1 (7)
Latino	16.4 (37)
Multiracial	5.8 (13)
White	58.4 (132)
Hispanic	
Hispanic	19.6 (44)
Not Hispanic	80.4 (180)
Education Level	
Completed HS or GED	7 (16)
Some College	10.4 (24)
Completed College	20.4 (47)
Some Masters Completed	6.5 (15)
Masters Completed	52.6 (121)
Completed PhD or equivalent	3 (7)
Annual Income	
<USD 20,000	2.27 (6)
USD 20,000–USD 40,000	32.4 (73)
USD 40,000–USD 60,000	41.8 (94)
USD 60,000–USD 80,000	14.2 (32)
USD 80,000+	8.9 (20)

**Table 2:** Final subscale comparison between ARTIC-45 and factors from the EFA.

ARTIC 45 Original Constructs	Constructs following Qual Content Analysis
Underlying causes of problem behavior and symptoms	Organizational support for provision of TIC
Responses to problem behavior and symptoms	Trauma theory
On-the-job behavior	Reflective functioning on the job
Self-efficacy at work	Worker efficacy
Reactions to the work	Relationships with clients
Personal support of TIC	Efficacy of TIC
System-wide support of TIC	Importance of consequences
	Beliefs about clients
	Secondary traumatic stress

## Data Availability

The deidentified data presented in this study are available on request from the corresponding author.
